# Supplementary material for: Gre Factors Are Required for Biofilm Formation in Salmonella enterica Serovar Typhimurium by Targeting Transcription of the csgD Gene
Source: Microorganisms. 2022 Sep 27;10(10):1921. doi: 10.3390/microorganisms10101921 (PMC9610925; doi:10.3390/microorganisms10101921)

Supplementary material

Manuscript:

Gre factors are required for biofilm formation in *Salmonella enterica* serovar Typhimurium by targeting transcription of the *csgD* gene.

by Tania Gaviria-Cantin, Andrés Felipe Vargas, Youssef El Mouali, Carlos Jonay Jiménez, Annika Cimdins-Ahne, Cristina Madrid, Ute Römling and Carlos Balsalobre

Figure S1: Alignment of the GreA proteins from the *E. coli* strain MG1655 (GreA MG1655) and from the *S*. Typhimurium strains ATCC14028 and SL1433 (GreA SALM).

Table S1: Strains and plasmids used in this study

Table S2: Primers used in this study

File S1: Information regarding construction of the *csgD::lacZ* chromosomal fusions.

```
GreA MG1655  1   MQAIPMTLRGAEKLREELDFLKSVRRPEIIAAIAEAREHGDLKENAEYHAAREQQGFCEG 60
                 MQAIPMTLRGAEKLREELDFLKSVRRPEIIAAIAEAREHGDLKENAEYHAAREQQGFCEG
GreA SALM    1   MQAIPMTLRGAEKLREELDFLKSVRRPEIIAAIAEAREHGDLKENAEYHAAREQQGFCEG 60

GreA MG1655 61   RIKDIEAKLSNAQVIDVTKMPNNGRVIFGATVTVLNLDSDEEQTYRIVGDDEADFKQNLI  120
                 RIKDIEAKLSNAQVIDVTKMPNNGRVIFGATVTVLNLD+DEEQTYRIVGDDEADFKQNLI
GreA SALM   61   RIKDIEAKLSNAQVIDVTKMPNNGRVIFGATVTVLNLDTDEEQTYRIVGDDEADFKQNLI  120

GreA MG1655 121 SVNSPIARGLIGKEEDDVVVIKTPGGEVEFEVIKVEYL  158
                 SVNSPIARGLIGKE+DDVVVIKTPGG+VE+EV+KVEYL
GreA SALM   121 SVNSPIARGLIGKEQDDVVVIKTPGGDVEYEVLKVEYL  158

Identities:153/158(97%), Positives:158/158(100%), Gaps:0/158(0%)
```

Figure S1. Alignment of the GreA proteins from the *E. coli* strain MG1655 (GreA MG1655) and from the *S*. Typhimurium strains ATCC14028 and SL1433 (GreA SALM). In yellow are labelled the mismatches and in green the residues 41 and 44.

Table S1. Strains and plasmids used in this study.

Strain	Relevant characteristics	Reference
SV5015	*Salmonella enterica* serovar Typhimurium SL1344 *his*$^{+}$	[1]
TGC-1	SV5015Δ*greA*	[2]
TGC-2	SV5015Δ*greB*	[2]
TGC-3	SV5015Δ*greA*Δ*greB*	[2]
UMR1	ATCC 14028 nal^{R}	[3]
TGC-43	UMR1Δ*greA*-km^{R}	This study
TGC-44	UMR1Δ*greB*-km^{R}	This study
TGC-45	UMR1Δ*greA*Δ*greB*	This study
MAE50	UMR1Δ*csgD*	[4]
MAE46	UMR1Δ*ompR*	[4]
MAE52	UMR1 P*csgD1*	[5]
TGC-51	MAE52 Δ*greA*	This study
TGC-52	MAE52 Δ*greB*	This study
TGC-50	MAE52 Δ*greA*Δ*greB*-km^{R}	This study
TGC-46	UMR1 $csgD_{+9}$::*lacZ* (km^{R})	This study
TGC-47	UMR1 Δ*greA*Δ*greB* $csgD_{+9}$::*lacZ* (km^{R})	This study
TGC-48	UMR1 $csgD_{+147}$::*lacZ* (km^{R})	This study
TGC-49	UMR1 Δ*greA*Δ*greB* $csgD_{+147}$::*lacZ* (km^{R})	This study
TGC-61	SV5015 *araBAD*-cm^{R} *araC*-km^{R}	[2]
	UMR1 *araBAD*-cm^{R} *araC*-km^{R}	This study
	UMR1 Δ*greA*Δ*greB* *araBAD*-cm^{R} *araC*-km^{R}	This study
**Strain**	**Relevant characteristics**	**Reference**
pBR322	ori pMB1, tc^{R} ap^{R}	[6]
pBRgreA	pBR322+$greA_{SV5015}$, ap^{R}	[2]
pBRgreB	pBR322+$greB_{SV5015}$, ap^{R}	[2]
pBRgreAgreB	pBR322+$greAgreB_{SV5015}$, ap^{R}	[2]
pHM1883	Ptrc expression vector, ori pGB2, spec^{R}	[7]
pHM1873	pHM1883+$greA_{MG1655}$	[2]
pHM1854	pHM1883+$greA_{MG1655}$(D41A, E44Y)	[2]
pTT68	PBAD-MCS-*lacZ*, ori RO1600/MB1 amp^{R}	[2]
pUTR*csgD*	pTT68+5'UTR*csgD*	This study
pKD4	oriR6K FRT km^{R} PS1 PS2 amp^{R}	[8]
pCP20	pSC101 ori^{TS} cI857 λPR flp amp^{R}	[8]
pKG136	oriR6K FRT *lacZY* +t_{his} km^{R}	[9]

References

1. Vivero, A.; Banos, R. C.; Mariscotti, J. F.; Oliveros, J. C.; Garcia-del Portillo, F.; Juarez, A.; Madrid, C. (2008). Modulation of Horizontally Acquired Genes by the Hha-YdgT Proteins in *Salmonella enterica* Serovar Typhimurium. Journal of Bacteriology, **2008**, 190, 1152–1156. doi.org/10.1128/JB.01206-07

2. Gaviria-Cantin, T.; El Mouali, Y.; Le Guyon, S.; Römling, U.; Balsalobre, C. Gre factors-mediated control of *hilD* transcription is essential for the invasion of epithelial cells by *Salmonella enterica* serovar Typhimurium. **2017**. PLoS Pathogens, 13. doi.org/10.1371/journal.ppat.1006312

3. Römling, U.; Bian, Z.; Hammar, M.; Sierralta, W. D.; Normark, S. Curli fibers are highly conserved between *Salmonella typhimurium* and *Escherichia coli* with respect to operon structure and regulation. **1998**. Journal of Bacteriology, 180, 722–731. doi.org/10.1128/JB.180.3.722-731.1998

4. Römling, U.; Rohde, M.; Olsén, A.; Normark, S.; Reinköster, J. AgfD, the checkpoint of multicellular and aggregative behaviour in *Salmonella typhimurium* regulates at least two independent pathways. **2000**. Molecular Microbiology, 36, 10–23. doi.org/10.1046/J.1365-2958.2000.01822.X

5. Römling, U.; Sierralta, W. D.; Eriksson, K.; Normark, S. Multicellular and aggregative behaviour of *Salmonella typhimurium* strains is controlled by mutations in the *agfD* promoter. **1998**. Molecular Microbiology, 28, 249–264. doi.org/10.1046/J.1365-2958.1998.00791.X

6. Bolivar, F.; Rodriguez, R. L.; Greene, P. J.; Betlach, M. C.; Heyneker, H. L.; Boyer, H. W.; Crosa, J. H.; Falkow, S. Construction and characterization of new cloning vehicles. II. A multipurpose cloning system. **1977**. Gene, 2, 95–113. doi.org/10.1016/0378-1119(77)90000-2

7. Vinella, D.; Potrykus, K.; Murphy, H.; Cashel, M. Effects on growth by changes of the balance between GreA, GreB, and DksA suggest mutual competition and functional redundancy in *Escherichia coli*. **2012**. Journal of Bacteriology, 194(2), 261–273. doi.org/10.1128/JB.06238-11

8. Datsenko, K.A.; Wanner, B.L. One-step inactivation of chromosomal genes in *Escherichia coli* K-12 using PCR products. **2000**. Proc Natl Acad Sci U S A. 97:6640–6645. doi: 10.1073/pnas.120163297

9. Ellermeier, C. D.; Janakiraman, A.; Slauch, J. M. Construction of targeted single copy lac fusions using lambda Red and FLP-mediated site-specific recombination in bacteria. **2002**. Gene. 290: 153–161. doi.org/10.1016/S0378-1119(02)00551-6

Table S2. Primers used in this study.

Primer name	Sequence
csgD +9	5'ACTTGCTTTAAGATTTGTAATGGCTAGATTGAAA ACAGTTAAAAGTGTAGGCTGGAGCTGCTTC3'
csgD+147	5'GGGGGCAGCTGTCAGATGTGCGATTAAAAAAAG TGGAGTTTCATCATGTAGGCTGGAGCTGCTTC3'
csgD lac Rev	5'GTAACTCTGCTGCTACAATCCAGGTCAGATAGC GTTTCATGGCCCATATGAATATCCTCCTTAGT3'
csgD up	5'CTTTAAGATTTGTAATGGC3'
csgD down	5'GCATGCAGGTTTCCGGTAGC3'
csgD UTR Fw	5'GCCCATGGCAGTTAAAAGTATTTTCG3'
csgDUTR Rv	5'GCGTCGACCATTAAACATGATGAAAC3'
qCsgDfw	5'ACGCTACTGAAGACCAGGAAC3'
qCsgDrev	5'GCATTCGCCACGCAGAATA3'
qrecAfw	5'GGCGAAATCGGCGACTCT3'
qrecArv	5'CATACGGATCTGGTTGATGAAAATC3'

File S1. Information regarding construction of the *csgD::lacZ* chromosomal fusions.

Nucleotide sequence of the SL1344 chromosomal region carrying the *csgD* gene. The first and last codon of the *csgD* ORF are indicated in bold. The bold capital G indicates the +1 position of the *csgD* transcript. The position of the *csgD* specific sequences of primers CsgD+9, CsgD+147 and CsgD lac Rev are indicated underlined in yellow, green and blue, respectively. The P1 and P2 sequence flanking the antibiotic resistance cassette are indicated in violet and red color, respectively.

```
   1 tggttacaag tttaacactt gctttaagat ttgtaatggc tagattgaaa acaGttaaaa
  61 gtattttcgt aaatattttt ctctttctgg ataatgggct atttcaaccc acagcagtgc
 121 aacatctgtc agtacttctg gtgcctttat tttatggggg cagctgtcag atgtgcgatt
 181 aaaaaaagtg gagtttcatc atgtttaatg aagtccatag tagtcatggt cacacactat
 241 tgttgatcac aaagccatct ctgcaagcta cggcattatt gcaacattta aagcaatcgc
     ...........................................................
 781 ttttcaaaaa gatagctgtc aaaaatcgca cccaggcagt ttcatgggca aacgataatc
 841 tcaggcggta aggccatgaa acgctatctg acctggattg tagcagcaga gttactgttc
 901 gctaccggaa acctgcatgc caatgaagtt gaagtcgagg ttcccggatt gttaaccgac
 961 cataccgtct cttcgatagg acatgaattc tatcgtgcat tcagcgacaa atgggaaagc
1021 gaatacaccg gcaatctgac cattaatgaa a
```

CsgD+9
5’ACTTGCTTTAAGATTTGTAATGGCTAGATTGAAAACAGTTAAAAGTGTAGGCTGGAGCTGCTTC3’
CsgD+147
5’GGGGGCAGCTGTCAGATGTGCGATTAAAAAAAGTGGAGTTTCATCATGTAGGCTGGAGCTGCTTC3’
CsgD lac Rev
5’GTAACTCTGCTGCTACAATCCAGGTCAGATAGCGTTTCATGGCCCATATGAATATCCTCCTTAGT3’

Below, in I to IV, a schematic representation of the generation of the *csgD*$_{+9}$*::lacZ* chromosomal fusion is shown.

Step I: PCR amplification of a fragment carrying an antibiotic resistance cassette flanked by two FRT sequences and *csgD* specific sequences (yellow and blue underlined sequences).

Step II: Integration of the PCR fragment into the SL1344 chromosome by homologous recombination.

Step III: Elimination of the antibiotic resistance cassette by site-specific recombination of the FRT sequences mediated by the FLP recombinase encoded by pCP20.

Step IV: Integration of the pKG136 plasmid carrying a promoter less *lacZ* gene downstream of a FRT sequence into the SL1344 chromosome.

Red framed panel are the final *csgD*$_{+9}$*::lacZ* and *csgD*$_{+147}$*::lacZ* chromosomal construct.

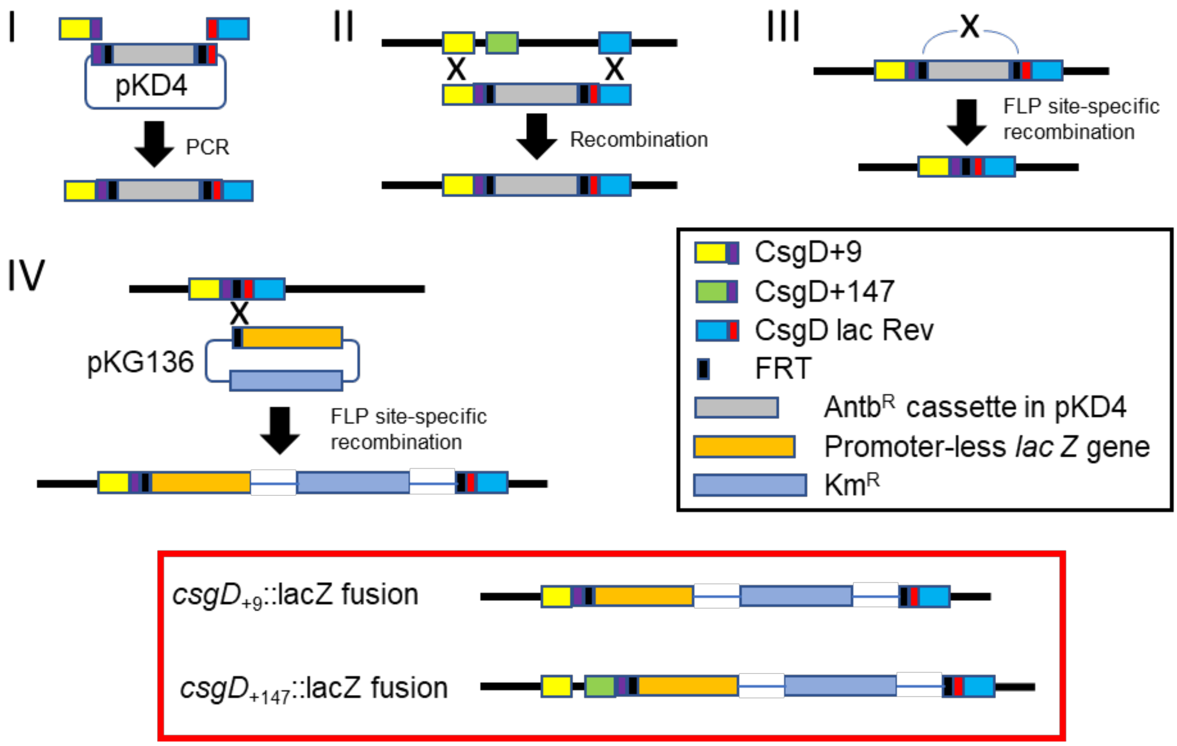

Supplement: Supplementary file 1 [file microorganisms-10-01921-s001.zip › microorganisms-1866021-supplementary.pdf]
